# A Robust and Fast Computation Touchless Palm Print Recognition System Using LHEAT and the IFkNCN Classifier

**DOI:** 10.1155/2015/360217

**Published:** 2015-05-31

**Authors:** Haryati Jaafar, Salwani Ibrahim, Dzati Athiar Ramli

**Affiliations:** Intelligent Biometric Group, School of Electrical and Electronic Engineering, Universiti Sains Malaysia Engineering Campus, 14300 Nibong Tebal, Penang, Malaysia

## Abstract

Mobile implementation is a current trend in biometric design. This paper proposes a new approach to palm print recognition, in which smart phones are used to capture palm print images at a distance. A touchless system was developed because of public demand for privacy and sanitation. Robust hand tracking, image enhancement, and fast computation processing algorithms are required for effective touchless and mobile-based recognition. In this project, hand tracking and the region of interest (ROI) extraction method were discussed. A sliding neighborhood operation with local histogram equalization, followed by a local adaptive thresholding or LHEAT approach, was proposed in the image enhancement stage to manage low-quality palm print images. To accelerate the recognition process, a new classifier, improved fuzzy-based *k* nearest centroid neighbor (IFkNCN), was implemented. By removing outliers and reducing the amount of training data, this classifier exhibited faster computation. Our experimental results demonstrate that a touchless palm print system using LHEAT and IFkNCN achieves a promising recognition rate of 98.64%.

## 1. Introduction

Palm print recognition has been widely investigated for the last decade in the field of pattern recognition. Similar to fingerprint recognition, palm print technology is based on the aggregate of information presented in a friction ridge impression. Although the image quality of a fingerprint is robust because of multiple lines, wrinkles, and ridges, a palm print includes even more information. A palm print covers a wider area than a fingerprint and contains characteristics such as palmar creases and triradius that are useful for recognition [1]. More importantly, ridge structures remain unchanged throughout life, except for a change in size [2]. A palm print is distinctive and thick, enabling easy capture by low-resolution devices. Therefore, palm print detection systems have a low cost and require minimum user cooperation for extraction [3]. Most palm print biometrics utilizes scanners or charge-coupled device (CCD) cameras as the input sensor [4, 5]. Because users must touch the sensor to acquire their hand images, users are concerned about hygiene, particularly in public areas, such as hospitals, malls, and streets [6, 7]. Disease-causing organisms, such as influenza virus, can be passed by indirect contact, and a susceptible individual can be infected from contact with a contaminated surface. The surface can become contaminated easily [6]; therefore, a touchless approach is required for palm print biometric technology.

The development of a touchless palm print recognition system is not straightforward. The hand position of the user during image acquisition is always changing. A touchless system does not require the user to touch or hold any platform or guidance peg. Users can open their hand, close their hand, or pose in a natural manner [6], and the hand can be deformed in other manners, including rotation, scale variability, and palm stretching, compared with touch-based systems [8]. Therefore, hand tracking and valley detection are challenging. As a result, hand tracking and region of interest (ROI) segmentation are difficult to implement. Complex backgrounds, poor ridge structures, and small image areas result in low-quality palm print images. The presence of noise/degradation (linear or nonlinear) and illumination changes [9] may reduce recognition accuracy. The computation times for the recognition process also must be considered. Because palm print systems consist of many major processes, such as data acquisition, preprocessing, feature extraction, and classification, fast processing algorithms are crucial [10, 11].

**Figure 1:**
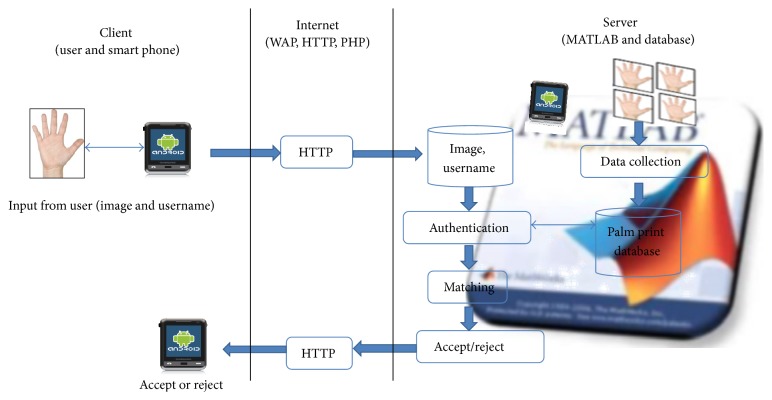
Overall research architecture.

This paper focuses on solutions for low-quality palm print images and computation times and includes a brief discussion of hand tracking and ROI segmentation. The overall research can be divided into three parts which are the client or smart phone side, internet side, and the server side which are illustrated as in Figure 1.

For the client side, the Android application for capturing biometric data is developed and it programs by using the latest few versions of Android OS, ranging from version 1.6 to version 4.1.2. Its programs support the mobile phone camera with the resolution up to 3.2 megapixels; hence only a few smart phones can be used for testing. Due to the existing camera application that varies for almost all smart phones and tablets, a customized camera application with the integration of enrolment and identification functions is developed for this research. The internet site is to connect the communication between smart phone and server and the connection is done via Wi-Fi and the PHP script is created to invoke the MATLAB program in the server.

The last part is server side where all the MATLAB programming including hand image identification, ROI extraction, palm print feature extraction, and pattern matching algorithms is written. The server software used in the project is free software where a personal computer serves as a server and has limited access from the client. Several palm print feature extraction algorithms which are based on subspace method are developed and evaluated for the fast and efficient mobile biometric system. Details of these operations can be found in Ibrahim and Ramli [12].

This study focused on the server side where two major contributions, that is, image enhancement and classification processes, have been developed to improve the quality of touchless palm print recognition systems. We propose a local histogram equalization and adaptive thresholding (LHEAT) technique for image enhancement. This technique is an improved version of the local histogram equalization (LHE) and local adaptive thresholding (LAT) techniques. Unlike previous methods [13–16], we used the sliding neighborhood operation for faster computation [17]. To accelerate the recognition process, the improved fuzzy-based *k* nearest centroid neighbor (IFkNCN) was used as the classifier for the system. The sliding neighborhood operation in the LHEAT technique also reduces the processing time of the image enhancement stage compared with the baseline LHE and LAT techniques.

This paper is organized as follows.  Section 2 presents related works and motivation. The proposed classifier for the palm print recognition system is described in Section 3. The experimental results are explained in Section 4, and Section 5 summarizes the work.

## 2. Related Works and Motivation

Many methods have been proposed to overcome the challenges associated with palm print recognition. Han and Lee [5] described two CMOS web cameras placed in parallel to segment the ROI of 1200 palm print images of identical size. The first camera captures the infrared image for hand detection, and the second camera is used to acquire the color image in normal lighting. The images are normalized using information on skin color and hand shape. The normalized images are then segmented to determine the ROI using the ordinal code approach and then classified with the Hamming distance classifier. Experimental results have shown that the equal error rate (EER) of the verification test is 0.54% and that the average acquisition time is 1.2 seconds. Feng et al. [18] used the Viola-Jones method [19] to detect the hand position after capturing 2000 images. In this study, images were acquired in different positions with various lighting and cluster backgrounds. Subsequently, a coarse-to-fine strategy was used to detect the key points on the hand. The key hand points were then verified with the shape context descriptor before the images were segmented into the ROI. The boosting classifier cascade [20] has previously been applied, and the accuracy rate was 93.8%, with a 178 ms average processing time for one image. Michael et al. [2] described a touchless palm print recognition system that was designed using a low-resolution CMOS web camera to acquire real-time palm print images. A hand tracking algorithm, that is, skin color thresholding and hand valley detection algorithm, was developed to automatically track and detect the ROI of the palm print. The Laplacian isotropic derivative operator was used to enhance the contrast and sharpness of the palm print feature, and a Gaussian low-pass filter was applied to smooth the palm print image and bridge some small gaps in the line. The modified probabilistic neural network (PNN) was used to classify the palm print texture. The accuracy rate was greater than 90%. Similar to previous studies, Michael et al. [21] used local-ridge-enhancement (LRE) to enhance the contrast and sharpness of images of both the right and left hands. The LRE was used to determine which section of the image contains important lines and ridge patterns and then amplify only those areas. The support vector machine (SVM) was used, and the average accuracy rates for the left and right hands were 97% to 98%, respectively.

Although previous researchers have achieved greater than 90% accuracy, the palm print image was captured in a semiclosed environment in a boxlike setup with an illumination source on top. This setup results in clean images with prefixed illumination settings [22]. The high accuracy is not reflective of the real environment. In the present study, an Android smart phone was used to capture the images, allowing users to easily access their system every day. Because the images were captured in the real environment, they were exposed to different levels of noises and blurring because of variations in illumination, background, and focus. Noise can also be due to bit errors in transmission or introduced during the signal acquisition stage.

We propose a touchless palm print recognition system that can manage real environment variability. The two areas discussed are image enhancement and classification. In image enhancement, a LHEAT technique was used. The purpose of LHE is to ensure that the brightness levels are distributed equally [15, 23]. In the LHE, the image is divided into small blocks or local *N* × *M* neighborhood regions. Each block or inner window is surrounded by a larger block or outer window, which is used to calculate the mapping function lookup for the inner window. To remove the borders of the block, the mapping function is interpolated between neighborhood blocks [15]. The LHE is an excellent image enhancement method. However, in the palm print image, considerable background noise and variation in contrast and illumination exist. Occasionally, the LHE overenhances the image contrast and causes degradation of the image [13, 14, 16]. Then, the binarization technique, LAT, is applied. In LAT, the threshold extracts the useful information from an image that has been enhanced by LHE and separates the foreground from the background with nonuniform illumination. Several methods, such as those described in Bersen, Niblack, Chow and Kaneko, and Sauvola [24], have been used to calculate the threshold values. Sauvola's method is most frequently used and was implemented here because of its promising results for degraded images.

In the pattern recognition system, there are two modes of recognition: verification and identification. This study focuses on the touchless palm print recognition system with identification mode. The identification mode is the time during which the system recognizes the user's identity by comparing the presented sample against the entire database to find a possible match [2]. Choosing the correct classification model becomes an important issue in palm print recognition to ensure that the system can identify a person in a short time. The *k* nearest neighbor (kNN) method is a nonparametric classifier widely used for pattern classification. This classifier is simple and easy to implement [25]. Nevertheless, there are some problems with this classifier; the performance of kNN often fails because of the lack of sample distribution information [26, 27] and not carefully assigning the class label before classification [28]. IFkNCN may resolve these limitations. This classifier incorporates centroid-based distance and fuzzy rule approaches with triangle inequality. The classifier removes the training samples that are far from the testing point or the query point by setting a threshold. The training samples that are located outside of the threshold are called outliers and defined as a noisy sample, which does not fit to the assumed class label for the query point. By removing the outliers, future processing focuses on the important training samples or candidate training samples, and this focus reduces the computational complexity in the searching stage. The query point is classified based on the centroid-distance and fuzzy rule system. The centroid-distance method is applied to ensure that the selected training samples are distributed sufficiently in the region of the neighborhood with the nearest neighbors located around the query point. Consequently, the fuzzy-based rule is used to solve the ambiguity of the weighting distance between the query point and its nearest neighbors.

## 3. Proposed Method

**Figure 2:**
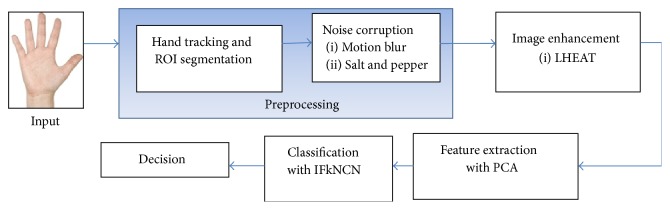
Block diagram of a touchless palm print recognition system.

Figure 2 displays the overall procedure for a touchless palm print recognition system.

In this work, a new comprehensive collection of palm print database was developed. This database currently was containing 2400 color images corresponding to 40 users who were Asian race students where each user had 60 palm print images. This database will be released to the public as benchmark data and it can be downloaded from the website of Intelligent Biometric Group (IBG), Universiti Sains Malaysia (USM), for research and educational purposes. All the users who are taking part in the data collection are completely voluntary and each volunteer gave verbal consent before collecting the image. The age of the user ranged from 19 to 23 years. An input image is acquired using a HTC One X Android mobile phone with 8 megapixels of image resolution and a stable background. The data collection is divided into 3 sessions; the first session is used for training purpose. The latter two sessions are used for testing purpose. The time interval for each session is in two weeks' time.

**Figure 3:**
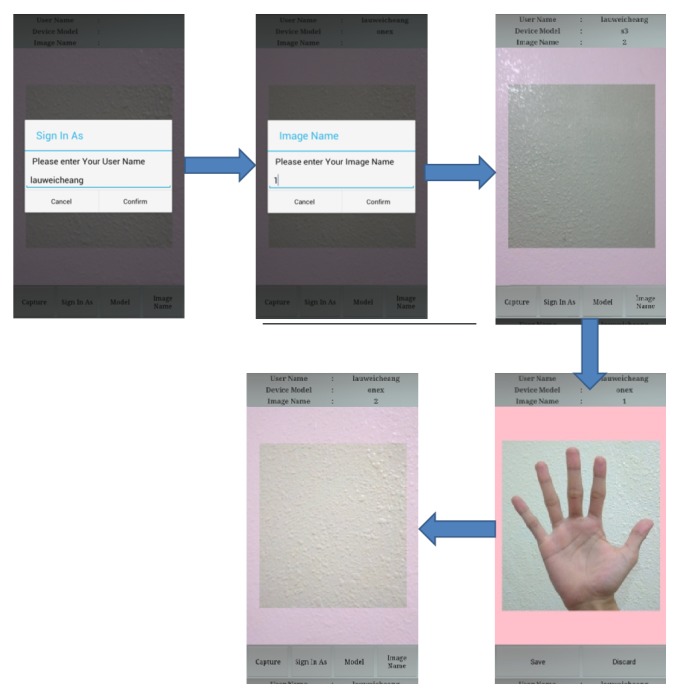
Data enrolment process.

For enrolment process, a user needs to follow the instruction displayed on the smart phone screen as shown in Figure 3. Firstly, the user was required to sign in and key in the image name. Subsequently, the users were simply asked to put their palm print naturally in front of the acquisition device. A semitransparent pink color box acts as a constraint box to ensure the palm and fingers lie inside the box. The pixels that lie outside of the constraint will be cropped. So the distance between hand and device is set as constant. Once the image was captured, it was saved into the database and this process was repeated for new image and user.

**Figure 4:**
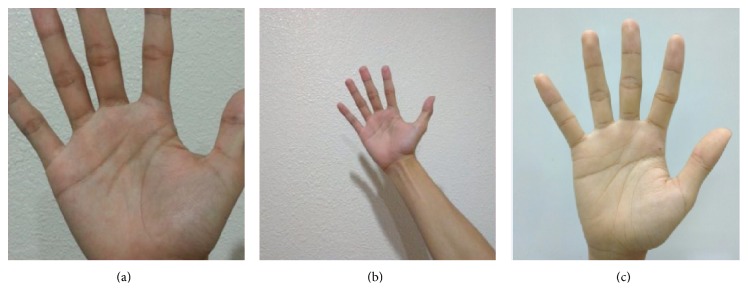
Hand image detection: (a) original RGB hand image; (b) binarized image. Hand position: (a) too close; (b) too far; and (c) suitable distance.

**Figure 5:**
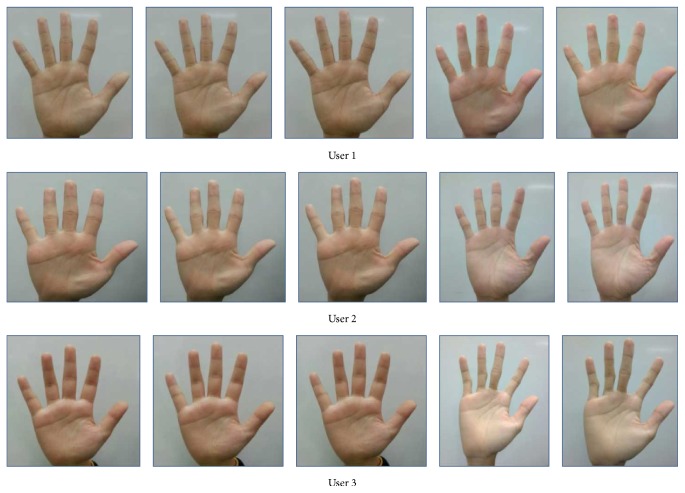
Original hand images captured by a smart phone camera for 5 different samples.

As no peg or other tool is used in the system, the users may place their hands at different heights above the mobile phone camera. The palm image appears large and clear when the palm is placed near the camera. Many line features and ridges are captured at near distance. However, if the hand is positioned too close to the mobile phone, the entire hand may not be captured in the image, and some parts of the palm print image may be occluded, as shown in Figure 4(a) [6]. When the hand is moved away from the camera, the focus fades, and some print information disappears (Figure 4(b)) [2]. The optimal distance between the hand and mobile phone is set according to the image preview in the enrolment process in Figure 3, enabling the whole hand image to be captured, as shown in Figure 4(c). Some examples of image of the whole palm print are shown in Figure 5.

The file were stored in JPEG format. Each folder was named as “S_x.” “S_x” represents the identity of the user which ranges from 1 to 40. Each folder had 60 palm print images. During preprocessing, the image was segmented to determine the ROI. This process is called hand tracking and ROI segmentation. The image was then corrupted by adding noises, such as motion blur noise and salt and pepper noise. Subsequently, the LHEAT method was applied to enhance the image. Then, feature extraction was performed. Principle analysis component (PCA) was employed to extract the image data and reduce the dimensionality of the input data. Finally, the image was classified by the IFkNCN classifier.

### 3.1. Preprocessing

**Figure 6:**
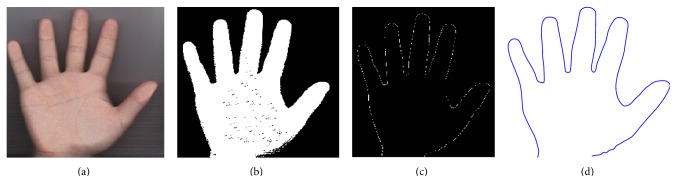
Hand image detection: (a) original RGB hand image; (b) binarized image; (c) hand contour with the Canny method; (d) perfect hand boundary plot.

There are three major steps in the hand tracking and ROI segmentation stage: hand image identification, peak and valley detection, and ROI extraction [12]. In the hand image identification step, the RGB image is transformed into a grayscale image and then converted to a binary image. Because the lighting conditions in the camera setup are uncontrolled, straightforward hand identification is not possible. Noise results in many small holes. The noise and unsmooth regions are removed by filling the small holes in the hand region. Once the noise is removed, the edge of the image is detected using the Canny edge detection algorithm. The hand boundary of the image is traced before the perfect hand counter is acquired, as shown in Figure 6.

**Figure 7:**
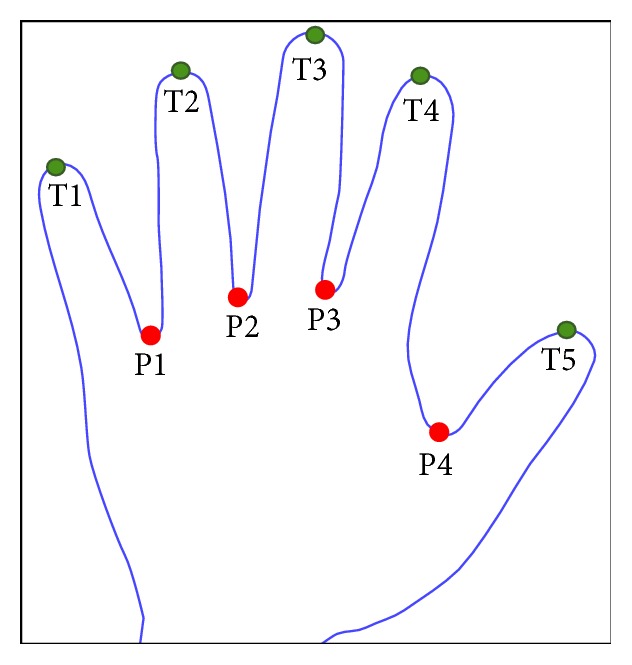
Five peaks and four valleys indicate the tips and roots of the fingers.

Because the image was captured without pegs or guiding bars, the palm print alignment varied in each collection. This variation caused the palm print image to be affected by rotation and may hamper accurate recognition. Therefore, the local minima and local maxima methods were used to detect peaks and valleys [29]. As shown in Figure 7, the peak and valley points in the hand boundary image were sorted and named before ROI segmentation.

**Figure 8:**
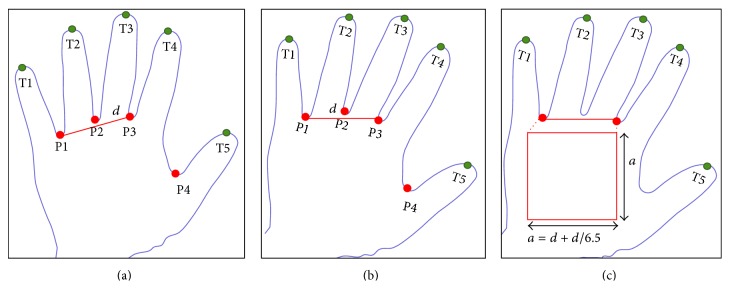
ROI segmentation process: (a) line drawn from P1 to P3; (b) rotated image; (c) ROI selection and detection.

The locations of three reference points, P1, P2, and P3, need to be detected in order to set up a coordinate system for palm print alignment. The size of ROI is dynamically determined by the distance between P1 and P3. It makes the ROI extraction scale invariant. To locate the ROI, a line was drawn between reference points; for example, P1 and P3 are shown in Figure 8(a) and labeled as “*d*.” The image was then rotated using a command “imrotate” in MATLAB function in order to ensure that the line was drawn horizontally as shown in Figure 8(b). The rotated image has the same size as the input image. A square shape was drawn, as shown in Figure 8(c), in which the length and width of the square were obtained as

(1) $$\begin{array}{c} a=d+\frac{d}{6.5}. \end{array}$$

The ROI was segmented, and the region outside the square was discarded. Then, the ROI was converted from RGB to grayscale.

**Figure 9:**
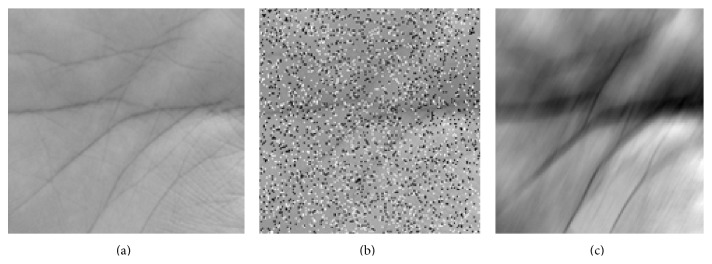
ROI image: (a) original; (b) degraded with salt and pepper noise; (c) degraded with motion blur noise.

To investigate the performance of the proposed method in noisy environments, the ROI image was corrupted using motion blur noise and salt and pepper noise, as shown in Figure 9. The level of source noise (*σ*) was set to 0.13.

### 3.2. Image Enhancement

Image enhancement is an important process that improves the image quality. Similar to the LHE and LAT methods, in the LHEAT method, the input image is broken into small blocks or local window neighborhoods that contain a pixel. In the LHEAT, the LHE is firstly obtained to ensure an equal distribution of the brightness levels. The LAT is employed to extract the useful information of the image that had been enhanced by the LTE and separated the foreground from the nonuniform illumination background. An input image is broken into small blocks or local window neighborhoods containing a pixel. This is similar in the LHE, LAT, and LHEAT. Each block is surrounded by a larger block. The input image is defined as *X* ∈ *R* ^*H*×*W*^, with dimensions of *H* × *W* pixels, and the enhanced image is defined as *Y* ∈ *R* ^*H*×*W*^, with *H* × *W* pixels. The input image is then divided into the block *T*_*i*_ = 1,…, *n* of window neighborhoods with the size *w* × *w*, where *w* < *W*, *w* < *H*, and *n* = [(*H* × *W*)/(*w* × *w*)].

**Figure 10:**
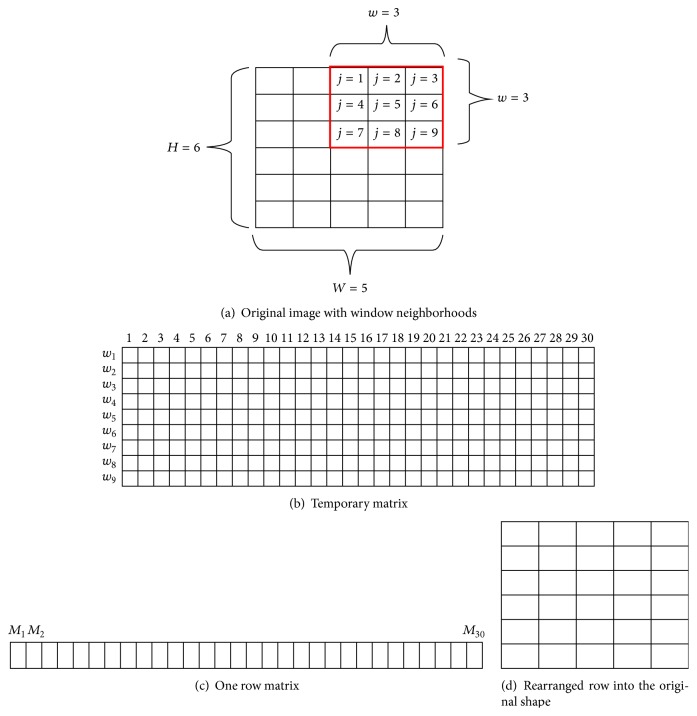
The sliding neighborhood operation.

Each pixel in the small block is calculated using a mapping function and threshold. The size of *w* should be sufficient to calculate the local illumination level, both objects, and the background [24]. However, this process results in a complex computation. To reduce the computation complexity and accelerate the computation, we used the sliding neighborhood operation [17].  Figure 10 shows an example of the sliding neighborhood operation. An image with a size of 6 × 5 pixels was divided into blocks of window neighborhoods with a size of 3 × 3 pixels. It is shown in Figure 10(a). The 6 × 5 image matrix was first rearranged into a 30-column (6 × 5 = 30) temporary matrix, as shown in Figure 10(b). Each column contained the value of the pixels in its nine-row (3 × 3 = 9) window. The temporary matrix was then reduced by using the local mean (*M*_*i*_):

(2) $$\begin{array}{c} M_{i}=\frac{1}{N}\overset{n}{\underset{j=1}{\sum }}w_{j}, \end{array}$$

where *w* was size of window neighborhoods, *j* was the number of pixels contained in each neighborhood, *i* was the number of columns in temporary matrix, and *N* was the total number of pixels in the block. After determining the local mean in (2), there was only one row left as shown in Figure 10(c). Subsequently, this row was rearranged into the original shape as shown in Figure 10(d).

There are three steps in the LHE technique: the probability density (PD), the cumulative distribution function (CDF), and the mapping function. The probability distribution of image PD for each block can be expressed as follows:

(3) $$\begin{array}{c} P\left(\;i\;\right)=\frac{n_{i}}{N}\;\text{for}\;i=0,1,\ldots ,L-1, \end{array}$$

where *n*_*i*_ is the input pixel number of level, *i* is the input luminance gray level, and *L* is gray level, which is 256.

Subsequently, the LHE uses an input-output mapping derived from CDF of the input histogram defined as follows:

(4) $$\begin{array}{c} C\left(\;i\;\right)=\overset{n}{\underset{i=0}{\sum }}P\left(\;i\;\right). \end{array}$$

Finally, the mapping function is determined from the CDF as follows:

(5) $$\begin{array}{c} g\left(\;i\;\right)=M+\left[\;\left(\;x_{i}-M\;\right)\times C\left(\;i\;\right)\;\right], \end{array}$$

where *M* is the mean value from (2).

Although the image has been enhanced, it remains mildly degraded because of the background noise and variation in contrast and illumination. The image was corrupted with two noises, motion blur noise and salt and pepper noise. The median filter, which has a 3 × 3 mask, was applied over the grayscale image. For an enhanced image, *g*(*i*), *q*(*i*) is the output median filter of length *l*, where *l* is the number of pixels over which median filtering takes place. When *l* is odd, the median filter is defined as follows:

(6) $$\begin{array}{c} q\left(\;i\;\right)=\text{median}\left\{\;g\left(\;i-k:i+k\;\right),\;\;k=\frac{\left(l-1\right)}{2}\;\right\}. \end{array}$$

When *l* is even, the mean of the two values at the center of the sorted sample list is used. The purpose of filtering is to reduce the effect of salt and pepper noise and the blur of the edge of the image.

Once the image has been filtered, the image is segmented using the LAT technique. The LAT separates the foreground from the background by converting the grayscale image into binary form. Sauvola's method was applied here, resulting in the following formula for the threshold:

(7) $$\begin{array}{c} T_{h}\left(\;i\;\right)=M\left[\;1+k\left(\;\frac{Z}{R}-1\;\right)\;\right], \end{array}$$

where *T*_*h*_ is the threshold, *k* is a positive value parameter with *k* = 0.5, *R* is the maximum value of the standard deviation, which was set at 128 for grayscale image, and *Z* is the standard deviation which can be found as

(8) $$\begin{array}{c} Z=\sqrt{\frac{1}{N-1}\overset{n}{\underset{j=1}{\sum }}\left(w_{j}-M\right)}. \end{array}$$

According to (8), the binarization results of Sauvola's method can be denoted as follows:

(9) $$\begin{array}{c} y\left(\;i\;\right)=\left\{\begin{array}{cc} 1 & \text{if}\;q\left(i\right)>T_{h}\left(i\right)\\ 0 & \text{otherwise.} \end{array}\right. \end{array}$$

**Figure 11:**
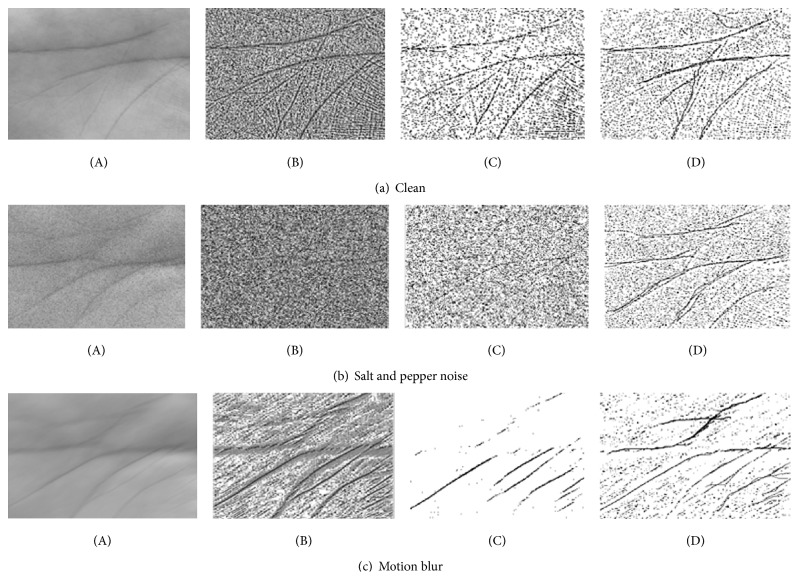
Comparison of image enhancement: (A) original image; (B) LHE; (C) LAT; and (D) LHEAT techniques.

Figure 11 shows the comparison of output results after applying the LHE and LHEAT techniques. The detail in the enhanced image using LHEAT was sharper, and fine details, such as ridges, were more visible.  Section 4.1 depicts the reduction in processing time and increased accuracy by applying the proposed image enhancement techniques.

### 3.3. Feature Extraction

Touchless palm print recognition must extract palm print features that can discriminate one individual from another. Occasionally, the captured images are difficult to extract because the line structures are discriminated individually. The creases and ridges of the palm cross and overlap one another, complicating the feature extraction task [30]. Recognition accuracy may decrease if the extraction is not performed properly.

In this paper, PCA was applied to create a set of compact features for effective recognition. This extraction technique has been widely used for dimensionality reduction in computer vision. This technique was selected because the features were more robust compared with other palm print recognition systems, such as eigenpalm [31], Gabor filters [32], Fourier transform [33], and wavelets [34].

The PCA transforms the original data from large space to a small subspace using a variance-covariance matrix structure. The first principle component shows the most variance while the last few principle components have less variance that is usually neglected since it has a noise effect.

Suppose a dataset {*x*_*i*_} where *i* = 1,2,…, *N* and *x*_*i*_ is rearranged in *P* ^2^ dimension. The PCA first computes the average vector of *x*_*i*_ and defined as

(10) $$\begin{array}{c} \overset{-}{x}=\frac{1}{N}\overset{n}{\underset{i=1}{\sum }}x_{i} \end{array}$$

whereas the deviations from *x*_*i*_ can be calculated by subtracting $\overset{-}{x}$:

(11) $$\begin{array}{c} \Phi_{i}=x_{i}-\overset{-}{x}. \end{array}$$

This step obtains a new matrix:

(12) $$\begin{array}{c} A=\left[\;\Phi_{1},\Phi_{2},\ldots ,\Phi_{n}\;\right]. \end{array}$$

That produces a dataset whose mean is zero. *A* is the *P* ^2^ × *N* dimensions.

Next, the covariance matrix is computed:

(13) $$\begin{array}{c} C=\overset{N}{\underset{i=1}{\sum }}\Phi_{i}{\Phi_{i}}^{T}=AA^{T}. \end{array}$$

However, (13) will produce a very large covariance matrix which is *P* ^2^ × *P* ^2^ dimensions. This causes the computation required to be huge and the system may slow down terribly or run out of memory. As a solution, the dimensional reduction is employed where the covariance matrix is expressed as

(14) $$\begin{array}{c} C=A^{T}A. \end{array}$$

Thus, the lower dimension of covariance matrix in *N* × *N* is obtained.

Next, the eigenvalues and eigenvectors of the *C* are computed. If the matrix *V* = (*V*_1_, *V*_2_,…, *V*_*p*_) contains the eigenvectors of a symmetric matrix *C*, then *V* is orthogonal, and *C* can be decomposed as

(15) $$\begin{array}{c} C=VDV^{T}, \end{array}$$

where *D* is a diagonal matrix of the eigenvalues and *V* is a matrix of eigenvectors. Then, the eigenvalues and corresponding eigenvectors are sorted in the order to decrease the dimensions. Finally, the optimum eigenvectors are chosen based on the largest value of eigenvalues. The details of these procedures can be found in Connie et al. [30].

### 3.4. Image Classification

**Figure 12:**
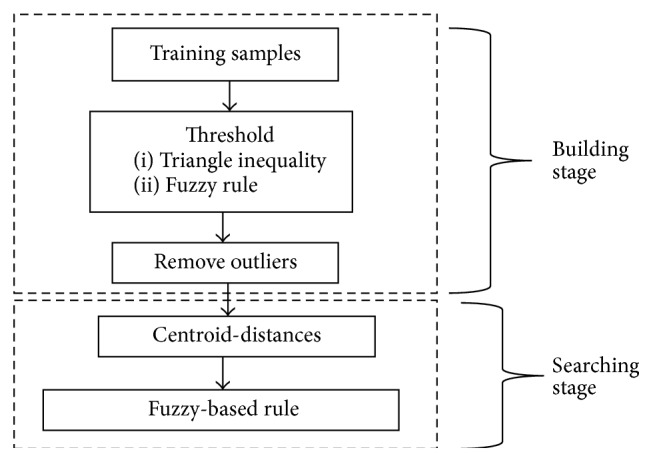
Architecture of the IFkNCN classifier.

This section describes the methods used for the IFkNCN classifier. There were two stages for this classifier: the building stage and the searching stage (Figure 12). In the building stages, triangle inequality and fuzzy IF-THEN rules were used to separate the samples into outliers and train candidate samples. For the searching stage, the surrounding rule was based on centroid-distance, and the weighting fuzzy-based rule was applied. The query point was classified by the minimum distances of the *k* neighbors and sample placement, considering the assignment of fuzzy membership to the query point.

*Building Stage*. In this stage, the palm print images were divided into 15 training sets and 40 testing sets. The distance of testing samples or query point and training sets was calculated, and the Euclidean distance was used.

Given a query point *y* and training sets *T* = {*x*_*j*_}_*j*=1_^*N*^, with *x*_*j*_ = {*c*_1_, *c*_2_,…, *c*_*M*_}, *N* is the number of training sets, *x*_*j*_ is the sample from the training sample, *M* is the number class, and *c* is the class label of *M*. The distance between the query point and training samples can be determined as follows:

(16) $$\begin{array}{c} d\left(\;y,x_{j}\;\right)=\sqrt{{\left(y-x_{j}\right)}^{T}\left(y-x_{j}\right)}, \end{array}$$

where *d*(*y*, *x*_*j*_) is the Euclidean distance, *N* is the number of training samples, *x*_*j*_ is the training sample, and *y* is the query point.

The distances were sorted in* ascending* order to determine the minimum and maximum distance. The threshold was set such that the training samples fell within a selected threshold distance and were considered inliers. Otherwise, they were considered to be outliers. To determine the threshold, triangle inequality was applied. The triangle inequality method requires that the distance between two objects (reference point and training samples; reference point and query point) cannot be less than the difference between the distances to any other object (query point and the training samples) [35]. More specifically, the distance between the query point and training samples satisfies the triangle inequality condition as follows:

(17) $$\begin{array}{c} d\left(\;y,x_{j}\;\right)\leq d\left(\;x_{j},z\;\right)+d\left(\;y,z\;\right), \end{array}$$

where *d*(*y*, *z*) is the distance from the query point to reference sample. In this study, the maximum distance obtained from (16) was assumed to be *d*(*y*, *z*). For faster computation, the distance between training sample and reference sample *d*(*x*_*j*_, *z*) was discarded. To eliminate the computation of *d*(*x*_*j*_, *z*), (17) was rewritten as follows:

(18) $$\begin{array}{c} 2d\left(\;y,x_{j}\;\right)\leq d\left(\;x_{j},z\;\right)+d\left(\;y,z\;\right). \end{array}$$

Because *d*(*y*, *x*_*j*_) ≤ *d*(*x*_*j*_, *z*), the value of *d*(*x*_*j*_, *z*) is not necessary, and (18) can be rearranged as follows:

(19) $$\begin{array}{c} d\left(\;y,x_{j}\;\right)\leq \frac{1}{2}d\left(\;y,z\;\right). \end{array}$$

**Figure 13:**
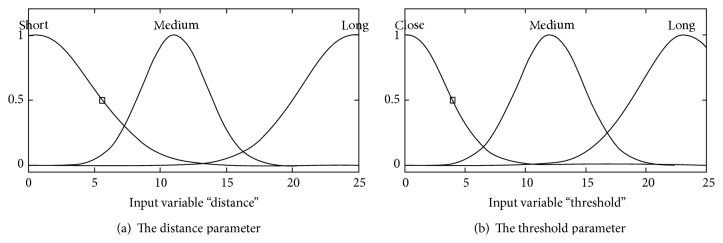
Input membership function.

The choice of threshold values is important because a large threshold value requires more computation. A small threshold makes the triangle inequality computation useless. To tackle the problem, the candidate outlier detection can be expressed by the fuzzy IF-THEN rules. Each input set was modeled by two functions, as depicted in Figure 13.

The membership functions were formed by Gaussian functions or a combination of Gaussian functions given by the following equation:

(20) $$\begin{array}{c} f\left(\;x,\sigma ,c\;\right)=e^{-{\left(x-c\right)}^{2}/2\sigma^{2}}, \end{array}$$

where *c* indicates the center of the peak and *σ* controls the width of the distribution. The parameters for each of the membership functions were determined by taking the best performing values using the development set [21].

**Figure 14:**
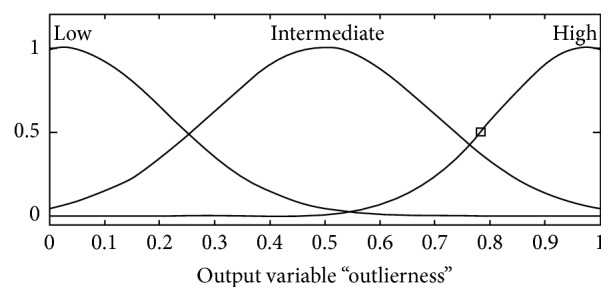
Output membership function.

The output membership functions were provided as Outlierness = {High, Intermediate, Low} and were modeled as shown in Figure 14. They have distribution functions similar to the input sets (which are Gaussian functions). The training sample was determined as an outlier if the distance of the training sample was long and the threshold was far and vice versa.

The Mamdani model was used to interpret the fuzzy set rules. This technique was used because it is intuitive and works well with human input. Nine rules were used to characterize the fuzzy rules. The main properties are as follows:

- If the distance is short and threshold is small, then outlierness is low.
- If the distance is short and threshold is large, then outlierness is intermediate.
- If the distance is long and threshold is small, then outlierness is intermediate.
- If the distance is long and threshold is far, then outlierness is high.

**Figure 15:**
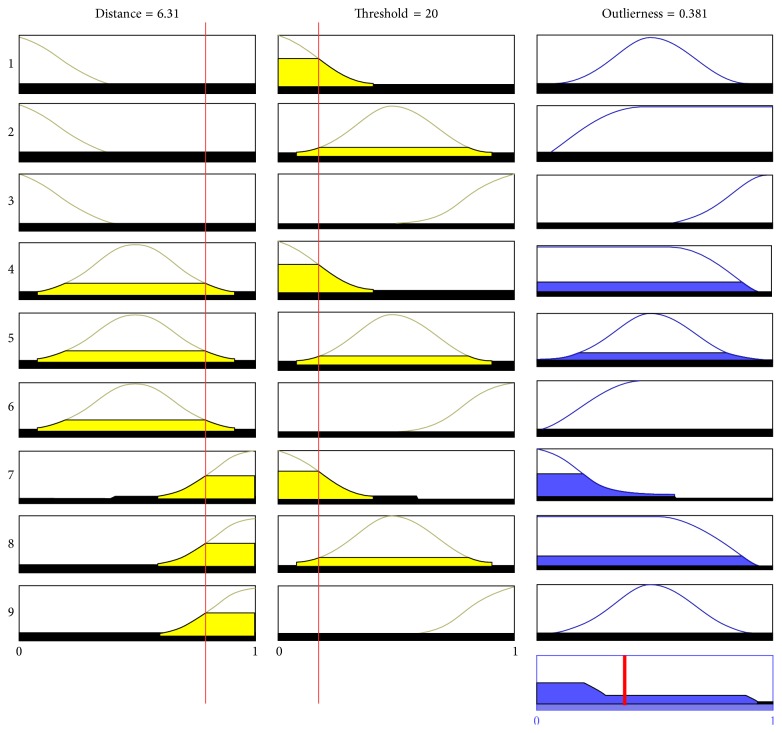
Example of the fuzzy IF-THEN rules.

The defuzzified output of the fuzzy procedure is influenced by the value of *d*(*y*, *x*_*j*_) and *d*(*y*, *z*). The fuzzy performance with a training sample with *d*(*y*, *x*_*j*_) = 6.31 and reference sample with *d*(*y*, *z*) = 20 is shown in Figure 15. The outlierness was 0.381, and the training sample was accepted as a candidate training sample. By removing the outlier, future processing only focuses on the candidate training samples.

*Searching Stage*. A surrounding fuzzy-based rule was proposed in which the rule is modified by the surrounding rule and the applied fuzzy rule. The main objective of this stage was to optimize the performance results while considering the surrounding fuzzy-based rules which are as follows:

- The *k* centroid nearest neighbors should be as close to the query point as possible and located symmetrically around the query point.
- The query point is classified by considering the fuzzy membership values.

Given a query point *y*, a set of candidate training samples *T* = {*x*_*j*_ ∈ *R* ^*m*^}_*j*=1_^*N*^, with *x*_*j*_ = {*c*_1_, *c*_2_,…, *c*_*M*_}, where *N* is the number of training samples, *x*_*j*_ is the training sample, *M* is the number of classes, and *c* is the class label of *M*, the procedures of the IFkNCN in building stage can be defined as follows:

(i) Select the candidate training sample as the first nearest centroid neighbor by sorting the distance of the query point and candidate training sample. Let the first nearest centroid neighbor be *x*_1_^NCN^.
(ii) For *k* = 2, find the first centroid of *x*_1_^NCN^ and the other candidate training samples are given as follows:
  (21) $$\begin{array}{c} {x_{2}}^{C}=\frac{{x_{1}}^{NCN}+x_{j}}{2}. \end{array}$$
(iii) Then, determine the second nearest centroid neighbors by finding the nearest distance of the first centroid and query point.
(iv) For *k* > 2, repeat the second step to find the other nearest centroid neighbors by determining the centroid between the training samples and previous nearest neighbors:
  (22) $$\begin{array}{c} {x_{k}}^{c}=\frac{1}{k}\overset{k}{\underset{i=1}{\sum }}{x_{j}}^{NCN}+x_{j}. \end{array}$$
(v) Let the set of *k* nearest centroid neighbors *T*_*jk*_^NCN^(*y*) = {*x*_*jk*_^NCN^ ∈ *R* ^*m*^}_*j*=1_^*k*^ and assign the fuzzy membership of the query point in every *k* nearest centroid neighbor. The fuzzy membership is as follows:
  (23) $$\begin{array}{c} {u_{i}}^{NCN}\left(\;y\;\right)=\frac{\sum_{j=1}^{k}u_{ij}\left(1/{\left\|y-{x_{jk}}^{NCN}\right\|}^{2/\left(m-1\right)}\right)}{\sum_{j=1}^{k}1/{\left\|y-{x_{jk}}^{NCN}\right\|}^{2/\left(m-1\right)}}, \end{array}$$
 where *i* = 1,2,…, *c*, *c* is the number of classes, *u*_*ij*_ is the membership degree of training sample, *x*_*jk*_ selected as the nearest neighbor, ‖*y* − *x*_*jk*_^NCN^‖ is the *L*-norm distance between the query point *x* and its nearest neighbor, and *m* is a fuzzy strength parameter, which is used to determine how heavily the distance is weighted when calculating each neighbor's contribution to the fuzzy membership values.
(vi) For the value of the fuzzy strength parameter, the value of *m* is set to 2. If *m* is 2, the fuzzy membership values are proportional to the inverse of the square of the distance, providing the optimal result in the classification process.
(vii) There are two methods to define *u*_*ij*_. One definition uses the crisp membership, in which the training samples assign all of the memberships to their known class and nonmemberships to other classes. The other definition uses the constraint of fuzzy membership; that is, when the *k* nearest neighbors of each training sample are found (say *x*_*k*_), the membership of *x*_*k*_ in each class can be assigned as follows:
  (24) $$\begin{array}{c} u_{ij}\left(\;x_{k}\;\right)=\left\{\begin{array}{cc} 0.51+0.49\left(\frac{n_{j}}{k}\right) & j=i\\ 0.49\left(\frac{n_{j}}{k}\right) & j\neq i, \end{array}\right. \end{array}$$
  where *n*_*j*_ denotes the number of neighbors of the *j*th training samples. The membership degree *u*_*ij*_ was defined using the constraint of fuzzy membership. The fuzzy membership constraint ensures that higher weight is assigned to the training samples in their own class and that lower weight is assigned to the other classes.
(ix) The query point to the class label can be classified by obtaining the highest fuzzy membership value:
  (25) $$\begin{array}{c} C\left(\;y\;\right)=\arg\max\left(\;{u_{i}}^{NCN}\left(y\right)\;\right). \end{array}$$
(x) Repeat steps (i) to (vii) for a new query point.

## 4. Experimental Results

As mentioned in Section 3, this study was conducted based on 2400 palm print images from 40 users. For each user, 15 images from the first session were randomly selected for training samples and the remaining 40 images from the second and third session were used as testing samples. Therefore, a total of 600 (15 × 40) and 1600 (40 × 40) images were used in the experiment. In order to gain an unbiased estimate of the generalization accuracy, the experiment was then run 10 times. The advantage of this method is that all of the test sets are independent and the reliability of the results can be improved.

Two major experiments, image enhancement and image classification, were conducted to evaluate the proposed touchless palm print recognition system. In the image enhancement experiment, three experiments were performed. The first experiment determined the optimal size of the window neighborhood for the LHEAT technique. The second experiment validated the usefulness of the image enhancement technique by comparing the results with and without applying the image enhancement technique. The third experiment compared the proposed LHEAT technique with the LHE [23] and LAT [24] techniques. In the image classification, the first experiment determined the optimal value of *k* and size of feature dimensions for the IFkNCN classifier and compared the performance of the IFkNCN with kNN [25], *k* nearest centroid neighborhood (kNCN) [27], and fuzzy kNN (FkNN) [28] classifiers.

The performance for both image enhancement and image classification experiments was evaluated based on processing time and classification accuracy (*C*_*A*_), where the *C*_*A*_ is defined as follows:

(26) $$\begin{array}{c} C_{A}=\frac{N_{C}}{N_{T}}\times 100\%, \end{array}$$

where *N*_*C*_ is the number of query points, which is classified correctly, and *N*_*T*_ is the total number of the query points.

All experiments were performed in MATLAB R2007 (b) and tested on Intel Core i7, 2.1 GHz CPU, 6 G RAM, and Windows 8 operating system.

### 4.1. Image Enhancement

To determine the optimal size of window neighborhood, *w* for the proposed method, a clean image was obtained, and the values of *w* were set to 3, 9, 15, and 19. The performance result was based on image quality and processing time. The results are shown in Table 1. The window neighborhood of *w* = 15 provided the best image quality. Although the image quality for *w* = 19 was similar to *w* = 15, the processing time was longer. Therefore, to size the window neighborhood, *w* = 15 was used in the subsequent experiments.

This section also validates the utility of the image enhancement techniques discussed in Section 3.2. In this experiment, the palm print features were extracted using PCA with a feature dimension size fixed at 80. Then, the IFkNCN classifier was obtained, in which the value of *k* was set to 5.  Table 2 shows the performance results with and without applying the image enhancement techniques. An improvement gain of approximately 3.61% in the *C*_*A*_ was achieved when the proposed image enhancement method was applied. Although the performance decreased because of degradation in image quality in the corrupted image, the image enhancement technique was able to recover more than 90% of the image compared with results without the image enhancement technique.

**Figure 16:**
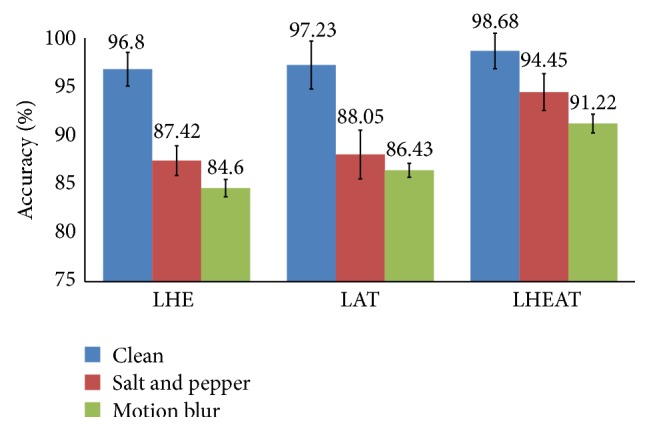
Performance of the LHE, LAT, and LHEAT methods for *C*_*A*_.

The next experiment investigated how the proposed LHEAT technique compared with previous techniques, such as LHE and LAT. The settings used in this experiment were the same as in the previous experiments. The result of the three experiments is shown in Figure 16. LHEAT performed better than LHE and LAT, yielding a *C*_*A*_ of more than 90% for the clean and corrupted images. LHE enhanced brightness levels by distributing the brightness equally and recovered original images that were over- and underexposed. When LAT was applied, the threshold changed dynamically across the image. LAT can remove background noise and variations in contrast and illumination. LHE and LAT in LHEAT complement one another and yield promising results.

**Figure 17:**
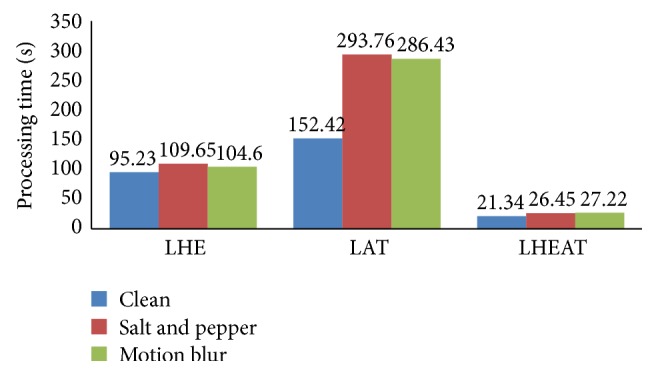
Performance of LHE, LAT, and LHEAT in processing time.

LHEAT gives another advantage over other methods in terms of its simplicity in computation. Normally, LHE and LAT require a time complexity of *O*(*w* ^2^ × *n* ^2^) with an image of size (*n* × *n*) with a size of window neighborhood (*w* × *w*). However, in the proposed LHEAT technique, the time complexity is *O*(*n* ^2^) because the sliding neighborhood is only used to obtain local mean (*M*) and local standard deviation (*Z*). Hence, the time required for LHEAT is much closer to global techniques.  Figure 17 shows a comparison of computation times during the image enhancement process. The LHEAT technique outperformed the LHE and LAT techniques.

### 4.2. Image Classification

Following the image enhancement experiments, the efficiency and robustness of the proposed IFkNCN classifier were evaluated. The first experiment in this section determined the optimal *k* value for the IFkNCN classifier. To avoid situations in which the classifier “ties” (identical number of votes for two different classes), an odd number for *k*, such as 1, 3, 5, 7, 9, 11, 13, 15, and 17, was used, and the size of feature dimension was fixed to 80. Comparison results are summarized in Table 3. IFkNCN achieved the highest *C*_*A*_ results when *k* was 5 and 7. The best *C*_*A*_ values were 98.54 ± 0.84%  (*k* = 5), 94.02 ± 0.54%  (*k* = 5), and 91.20 ± 1.10%  (*k* = 7) for clean, salt and pepper noise, and motion blur images, respectively. Because there was only a 0.12% difference between *k* = 7 and *k* = 5 for IFkNCN in motion blur images, the value of *k* is set to 5 to ease the calculation in the subsequent experiments. The results also showed that increasing the value of *k* further lowers the *C*_*A*_. When *k* increases, the number of nearest neighbors of the query point also increases. In this situation, some training samples from different classes, which have similar characteristics, were selected as the nearest neighbor, and these training samples were defined as overlapping samples. Misclassification often occurs near class boundaries in which an overlap occurs.

The second experiment determined the optimal feature dimension size for the IFkNCN classifier. The *k* value was set to 5, and the size of the feature dimension was set to 20, 60, 80, 100, and 120. The results are shown in Table 4. As expected, the palm print recognition achieved optimal results when the size of the feature dimension was set to 120. However, the value also had the highest processing time. When the feature dimension was set to 100, the processing time was reduced twofold lower than the feature dimension of 120. The difference in *C*_*A*_ between the 100 and 120 feature dimensions was relatively small (approximately 0.10%). Therefore, a feature dimension of 100 was selected as the optimal value for IFkNCN, and this size was used for the next experiment.

**Figure 18:**
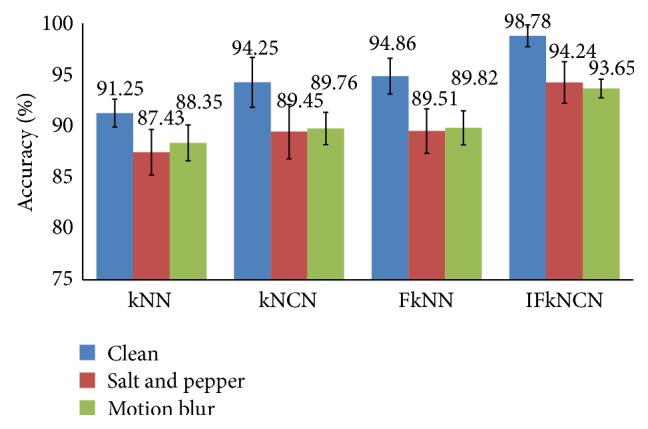
Comparison of IFkNCN with kNN, kNCN, and FkNN classifiers based on *C*_*A*_.

The subsequent experiment evaluated the proposed classifier. A comparison of IFkNCN with other previous nearest neighbor classifiers, such as kNN, kNCN, and FkNN, was performed. The optimal parameter values, that is, *k* = 5 and a feature dimension of 100, were used. The overall performance results based on *C*_*A*_ are described in Figure 18. By utilizing the strength of the centroid neighborhood while solving the ambiguity of the weighting distance between the query point and its nearest neighbors, the IFkNCN classifier outperformed the kNN, kNCN, and FkNN classifiers. The *C*_*A*_ of the IFkNCN increased approximately 7.53%, 6.81%, and 5.3% in the clean, salt and pepper, and motion blur images, respectively, compared with kNN, kNCN, and FkNN.

**Figure 19:**
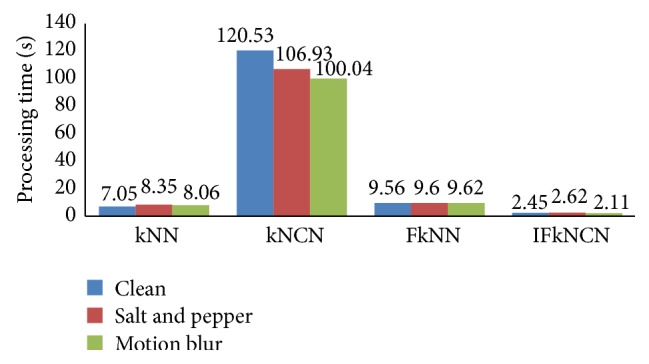
Comparison of IFkNCN with the kNN, kNCN, and FkNN classifiers based on processing time.

In addition to better accuracy, the proposed IFkNCN classifier also had better processing times in all conditions, as shown in Figure 19. By using the triangle inequality and fuzzy IF-THEN rules, the training samples that were not relevant to additional processing were removed. Accuracy did not decrease, but the processing time was 2.39 s, whereas the processing times for kNN, kNCN, and FkNN were 7.82 s, 109.17 s, and 9.59 s, respectively.

**Figure 20:**
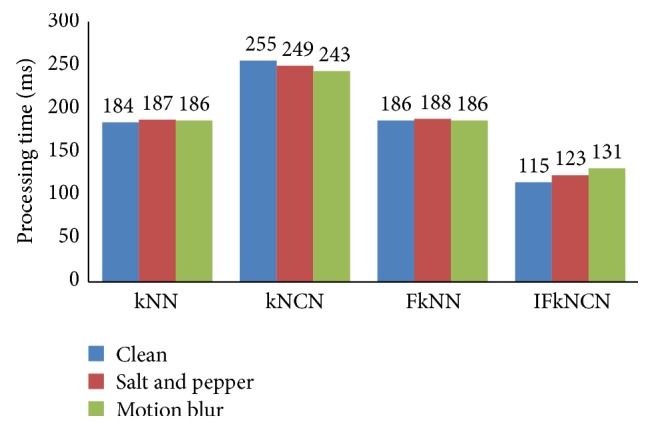
Processing speed of a touchless palm print system.

The time required to execute each process, that is, image preprocessing, image enhancement, feature extraction, and image classification, in the touchless palm print recognition is shown in Figure 20. The reported time is the average time required to process an input image from a user. The total time to identify a user was less than 130 ms. The speed demonstrated by the proposed system demonstrates that it has the potential for implementation in real-world applications.

## 5. Conclusions and Future Works

This paper presents a touchless palm print recognition method using an Android smart phone. The proposed system is accessible and practical. In addition, the device is cost-effective and does not require expensive hardware. This paper focused on image enhancement and image classification. To enhance the quality of the acquired images, we propose the LHEAT technique. Because the sliding neighborhood operation is applied in the LHEAT technique, the computation was much faster compared with previous techniques, such as LHE and LAT. The proposed technique was also able to reduce noise and increase the dominant line edges in the palm print image. Moreover, this method works well in noisy environments. This paper also presents a new type of classifier, called IFkNCN, that has advantages compared with the kNN classifier. The major advantage of the IFkNCN classifier is that it can remove the outliers and that its computation is efficient. Extensive experiments were performed to evaluate the performance of the system in terms of image enhancement and image classification. The proposed system exhibits promising results. Specifically, the *C*_*A*_ with the LHEAT technique was more than 90%, and the processing time was threefold lower than with the LHE and LAT methods. In addition, the *C*_*A*_ achieved by the IFkNCN method was improved to more than 90% for clean and corrupted images, and the processing time was less than 120 ms, which was substantially less compared with the other tested classifiers. The proposed touchless palm print system is convenient and able to manage real-time recognition challenges, such as environmental noise and lighting changes.

Although the purpose of this research has been achieved, there are some aspects that need to be taken into consideration for future work. Firstly, in order to ensure the development of touchless palm print system is more applicable in real application, experiment in various types of noises needs to be extracted before the ROI extraction. So the filtered process can be improved before the subsequent process is applied. Secondly, additional algorithms in the image enhancement can be added to improve the LHEAT performance, especially when the image is captured in various types of illumination, background, and focus. However, addition of other algorithms may slow down the speed of this technique. Thus, this problem should be considered if the online or real-time processing algorithm is required. For the classification process, the code optimization could be conducted to increase the computational efficiency of the IFkNCN classifier during the searching stage. Since the complexity of each training sample in searching stage is high, the code optimization process will be beneficial in offering better solution to overcome this complexity problem.

## Figures and Tables

**Table 1:** Performance with different sizes of the window neighborhood.

*w*	3	11	15	19
Image	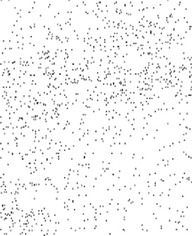	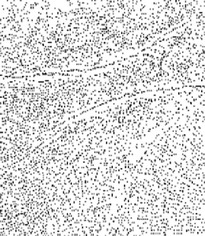	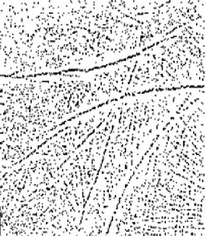	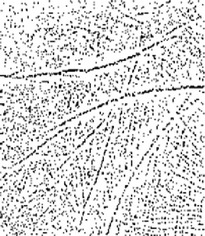

Time (s)	0.07	0.84	1.09	2.30

**Table 2:** Comparison of the image enhancement techniques.

Method	C_*A*_ (%)		
	Clean	Salt and pepper noise	Motion blur noise
Without image enhancement	96.40 ± 1.14	86.40 ± 2.07	88.80 ± 1.48
With LHEAT technique	98.42 ± 0.55	90.40 ± 0.89	93.60 ± 0.89

**Table 3:** Comparison of the CA results for different *k* values (results are in %).

Image	*k* = 1	*k* = 3	*k* = 5	*k* = 7	*k* = 9	*k* = 11	*k* = 13	*k* = 15	*k* = 17
Clean	96.02 ± 1.14	96.35 ± 0.95	**98.54 ± 0.84**	98.12 ± 0.98	97.67 ± 1.16	96.58 ± 1.24	96.34 ± 0.64	96.82 ± 1.14	96.34 ± 1.02
Salt and pepper	91.12 ± 0.82	93.54 ± 1.26	**94.02 ± 0.54**	93.84 ± 0.96	93.21 ± 1.12	93.15 ± 1.45	93.02 ± 0.98	92.34 ± 1.26	91.89 ± 0.66
Motion blur	88.02 ± 1.34	89.72 ± 1.22	91.08 ± 0.98	**91.20 ± 1.10**	90.33 ± 0.88	89.78 ± 0.45	89.54 ± 0.66	88.96 ± 1.82	89.02 ± 1.82

**Table 4:** Comparison of IFkNCN of different feature dimension values.

Dim.	Clean		Salt and pepper		Motion blur	
	Time (s)	C_*A*_ (%)	Time (s)	C_*A*_ (%)	Time (s)	C_*A*_ (%)
20	0.65	93.32 ± 1.22	0.74	91.50 ± 2.01	0.99	89.62 ± 1.52
40	0.86	93.56 ± 1.00	0.83	92.06 ± 1.88	1.03	90.12 ± 0.94
60	1.17	95.34 ± 0.94	1.15	92.95 ± 1.05	1.64	90.95 ± 1.32
80	1.54	98.64 ± 1.26	1.44	93.67 ± 1.22	1.71	91.02 ± 0.98
100	**1.32**	**98.96 ± 0.55**	**1.46**	**94.11 ± 1.14**	**1.92**	**92.45 ± 1.14**
120	5.43	99.02 ± 1.25	4.98	94.21 ± 1.35	5.24	92.49 ± 1.32
